# Mitochondrial Reactive Oxygen Species: Double-Edged Weapon in Host Defense and Pathological Inflammation During Infection

**DOI:** 10.3389/fimmu.2020.01649

**Published:** 2020-08-14

**Authors:** Prashanta Silwal, Jin Kyung Kim, Young Jae Kim, Eun-Kyeong Jo

**Affiliations:** ^1^Department of Microbiology, Chungnam National University School of Medicine, Daejeon, South Korea; ^2^Infection Control Convergence Research Center, Chungnam National University School of Medicine, Daejeon, South Korea

**Keywords:** mitochondrial ROS, infection, inflammation, immunity, host defense

## Abstract

Mitochondria are inevitable sources for the generation of mitochondrial reactive oxygen species (mtROS) due to their fundamental roles in respiration. mtROS were reported to be bactericidal weapons with an innate effector function during infection. However, the controlled generation of mtROS is vital for the induction of efficient immune responses because excessive production of mtROS with mitochondrial damage leads to sustained inflammation, resulting in pathological outcomes such as sepsis. Here, we discuss the beneficial and detrimental roles of mtROS in the innate immune system during bacterial, viral, and fungal infections. Recent evidence suggests that several pathogens have evolved multiple strategies to modulate mtROS for their own benefit. We are just beginning to understand the mechanisms by which mtROS generation is regulated and how mtROS affect protective and pathological responses during infection. Several agents/small molecules that prevent the uncontrolled production of mtROS are known to be beneficial in the maintenance of tissue homeostasis during sepsis. mtROS-targeted approaches need to be incorporated into preventive and therapeutic strategies against a variety of infections.

## Introduction

Mitochondria are essential organelles for the generation of reactive oxygen species (ROS) through respiration and function as a crucial signaling platform for various biological responses, including metabolism, innate immunity, and inflammation (1). Innate immune cells such as macrophages and neutrophils produce and employ mitochondrial reactive oxygen species (mtROS) as direct antimicrobial agents in host defense to combat pathogens (2). Accumulating evidence has revealed more complex molecular functions of mtROS in the activation of nucleoside oligomerization domain-, leucine-rich repeat-, and pyrin domain-containing protein 3 (NLRP3) inflammasomes and the regulation of innate signaling pathways triggered by numerous pattern-recognition receptor engagement (3, 4). Indeed, mtROS are critically required for innate host defense as effectors through their toxic action against pathogens. However, uncontrolled regulation of mtROS may lead to chronic inflammation and pathologies during infection (5, 6).

In this review, we discuss recent advances in our understanding of the protective role of mtROS associated with host-defensive signaling pathways involved in actions against pathogens during infection. We further highlight how pathogens evade mtROS-dependent antimicrobial responses or enhance mtROS-mediated pathological inflammation. In addition, we review the detrimental functions of mtROS when they are produced in excess in damaged cells and tissues during infection. We also introduce the idea that several agents/approaches evolved to modulate mtROS in the maintenance of tissue homeostasis in the context of sepsis. An understanding of the collective actions of mtROS in innate immune functions represents a new frontier in the development of novel therapeutic strategies against acute and chronic infections.

### Protective Functions of mtROS in the Activation of the Host Defense

#### mtROS in Innate Immune Signaling

A decade ago, strong evidence indicated that mtROS provide antimicrobial responses in the context of innate immunity. Numerous studies showed that the maximal bactericidal activity in innate immune cells depended on mtROS generation. Toll-like receptor (TLR; TLRs 1, 2, and 4) signaling triggers the recruitment of mitochondria to the phagosomes and augments mtROS generation to enhance macrophage bactericidal activity (7). Additional studies showed that the mammalian STE20-like protein kinase-1/2 (MST1/2) were required for ROS production through mitochondrial trafficking to the phagosomes, and that they promoted TLR-mediated assembly of the tumor necrosis factor receptor-associated factor 6 (TRAF6), an evolutionarily conserved signaling intermediate in Toll pathway (ECSIT) complex, thus enhancing antibacterial killing and inflammatory signaling in macrophages (7–9). Importantly, ECSIT plays a role in the assembly and activity of respiratory complex-I of the electron transport chain (ETC) to produce mtROS in macrophages after TLR4 stimulation (10). In neutrophils, mtROS mediates the functional responses such as oxidative burst, degranulation and apoptosis (11), as well as NETosis induced by Ca^2+^ ionophore where mitochondrial permeability transition pore (mPTP) is critically involved in mtROS production (12). These studies suggest the role for mtROS in the regulation of different aspects of innate immune responses in macrophages and neutrophils against pathogenic stimuli.

#### mtROS in Antibacterial and Antiparasitic Defense

Several other studies showed the role of mtROS as the antimicrobial components in the innate defense against bacterial infection. Early studies showed that Th1 cytokine interferon (IFN)-γ signaling activated transcriptional activation of the mitochondrial respiratory chain machinery through the nuclear receptor estrogen-related receptor α (ERRα; NR3B1), which is required for mtROS generation to promote clearance of *Listeria monocytogenes* (13). A more recent study revealed that ERRα is essentially required for antimicrobial host defense against *Mycobacterium tuberculosis* (Mtb) infection through autophagy activation, although whether mtROS was implicated in the activation of xenophagy, a selective autophagy-targeting pathogen (14), was not clarified (15). As mtROS play key roles in the maintenance of cellular homeostasis, such as autophagy (16, 17), additional studies are warranted to elucidate the roles of mtROS in the activation of xenophagy in the context of the innate immune defense. Although relatively uncharacterized in the function of mtROS in terms of antiparasitic defense, mtROS contributed to upregulating the intracellular clearance of *Leishmania donovani*, an intracellular parasite (18, 19).

Although there are some debates, the metabolic sensor 5' AMP-activated protein kinase (AMPK) inhibits the generation of mtROS, whereas hypoxia-inducible factor (HIF)-1α upregulates mtROS production. Mutual regulation between AMPK and HIF-1α is required to maintain mtROS at the optimal level, thereby promoting the innate defense against several pathogenic bacteria (20, 21). HIF-1α and the mammalian target of the rapamycin (mTOR) pathway are critical for driving an immunometabolic signaling toward glycolysis during infection (22, 23). The mTOR-mediated aerobic glycolysis in human monocytes (CD14^+^CD16^−^) results in the accumulation of ROS and inflammatory signaling to activate monocyte function (23). However, mTOR inhibition upregulates mtROS production and NLRP3 inflammasome activation to suppress the replication of *Trypanosoma cruzi* in macrophages (24). More investigation into the paradoxical function of mTOR signaling is required for a better understanding of its role in the coordination of immunometabolism and mtROS-related host defense. Metformin, a widely used antidiabetic drug, was beneficial in the clearance of *Legionella pneumophila* infection through AMPK signaling activation and mtROS generation (25). Metformin-mediated antibacterial effects were ameliorated by glutathione treatment, suggesting a protective role of mtROS in improving antibacterial immunity against *L. pneumophila* pneumonia (25). In addition, it should be noted that ERRα is regulated at the downstream of AMPK to enhance antimycobacterial immunity and plays a crucial role in the activation of autophagy (15). Therefore, a delicate control of immunometabolic shifting between aerobic glycolysis (HIF-1α/mTOR-mediated) and mitochondrial respiration (AMPK mediated) may affect the innate effector functions, at least partly through the regulation of mtROS generation.

Moreover, macrophage recognition of live bacteria led to a transient shift in the mitochondrial respiratory system and destabilization of the ETC complex I but increased the activity of complex II, which was essential for the antimicrobial response (26). During live bacterial sensing in macrophages, nicotinamide adenine dinucleotide phosphate (NADPH) oxidase-mediated ROS, in cooperation with mtROS, play essential roles in the early induction of ETC adaptations (26). In this regard, mtROS, which are derived from several respiratory complexes (complexes I, II, and III) (27, 28), may contribute to the different aspects of antimicrobial and inflammatory function, and further investigation of their specific roles depending on differential sources and cell types is warranted.

#### mtROS in Antiviral Signaling

Early studies highlighted the function of mtROS in the amplification of mitochondrial antiviral signaling (MAVS) and RIG-I-like receptor (RLR)-mediated antiviral responses (29). During infection with influenza A virus (IAV), both mitochondrial and dual oxidase (Doux) 2-generated ROS were essential for the antiviral host defense in normal human nasal epithelial (NHNE) cells. Notably, the induction of the IFN-λ gene and protein expression, which was mediated through ROS, contributed to the suppression of IAV viral titers (30). Another study showed that mtROS generation was required for IAV-induced signal transducer and activator of transcription (STAT) phosphorylation and IFN-stimulated gene expression, which promoted antiviral responses in NHNE cells (31). Scavenging mtROS led to the attenuation of innate immune responses to IAV and increased viral titers in nasal epithelial cells (30, 31). These findings suggest that mtROS play a crucial role in the induction of antiviral immune defense against IAV infection.

A recent study identified tetrachlorodibenzo-p-dioxin (TCDD)-inducible poly(ADP ribose) polymerase (TIPARP), a zinc finger antiviral protein (ZAP), as a pattern-recognition receptor for the RNA of Sindbis virus (32), a positive-sense single-stranded RNA virus belonging to the genus Alphavirus in the family Togaviridae (33). TIPARP was found to protect mice against lethal Sindbis virus infection through promotion of antiviral responses. It was noted that TIPARP redistribution from the nucleus to the cytoplasm during infection is mediated through mtROS-dependent oxidization of the nuclear pore and mitochondrial damage (32). This cytoplasmic accumulation of TIPARP, which is triggered by mtROS, seems to favor the persistence of TIPARP localization in the cytosol to mediate antiviral response in the host cells (32). In addition to TIPARP, there are several zinc finger domain-containing protein family members that participate in the inhibition of viral replication, particularly in HIV-1 and flavivirus infections (34, 35). The role of mtROS in most zinc finger domain-containing family members should be characterized in a future study.

mtROS also cooperate with several innate effectors/pathways in macrophages and/or other immune cells during infection. mtROS participate in the activation of the NLRP3 inflammasome, which plays a crucial function in the innate immune defense against numerous pathogens (3, 4). Indeed, mtROS activation serves as an important second signal to trigger NLRP3 inflammasome activation, which leads not only to the host defense against diverse bacterial, viral, and fungal infections but also to pathophysiological responses when uncontrolled or dysregulated (3). Elucidating the potential mechanisms of the coordinated mtROS generation could help develop further knowledge on host defense through appropriate activation of NLRP3 inflammasome complex during infections by various pathogens.

**Figure 1:**
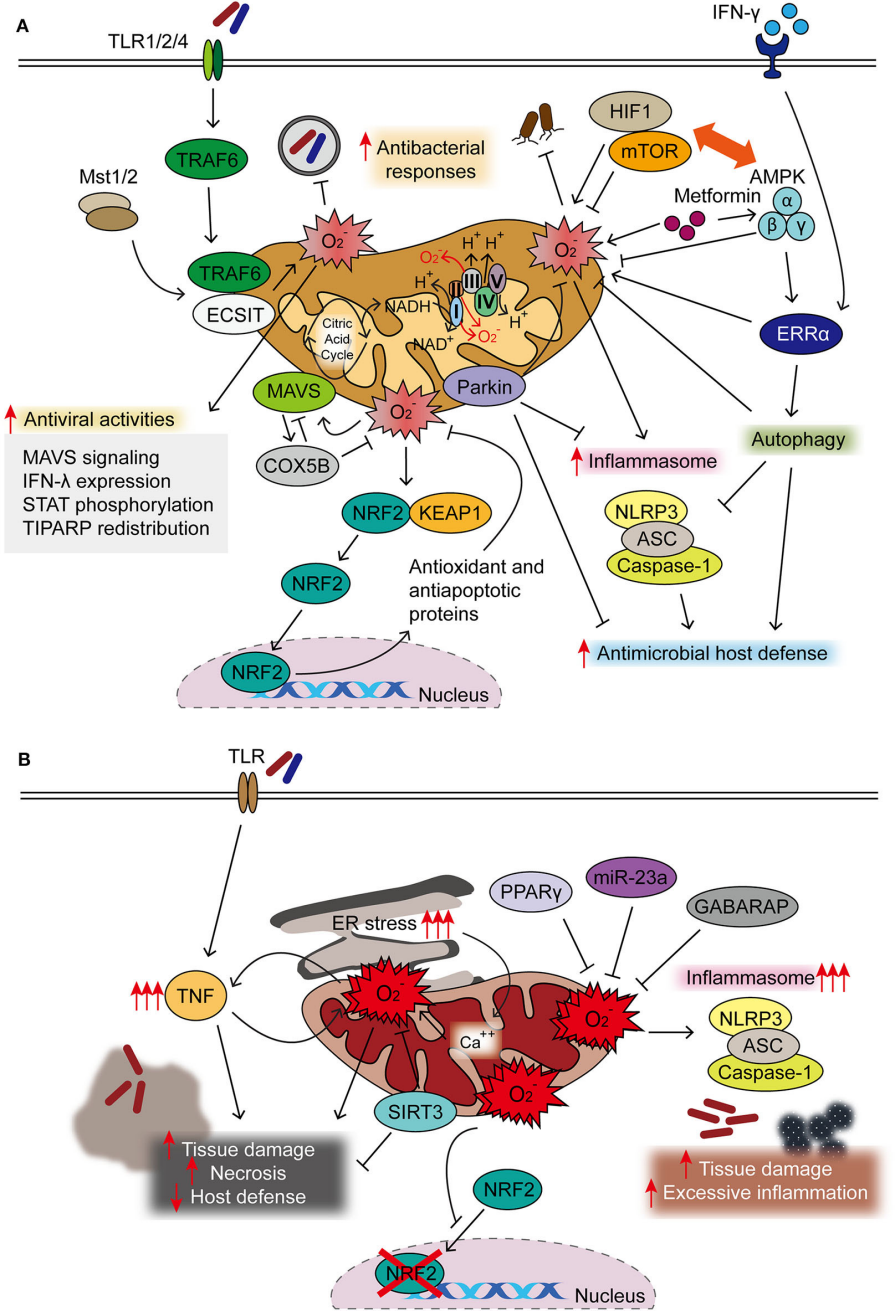
Protective and detrimental roles of mitochondrial reactive oxygen species (mtROS) during infection. **(A)** Majority of mtROS are generated by oxidative phosphorylation (OXPHOS) complexes I and III in macrophages activated through TLR signaling, interferon (IFN)-γ, and microbial infection. Both Mst1 and Mst2 promoted Toll-like receptor (TLR)-mediated assembly of the tumor necrosis factor receptor-associated factor 6–evolutionarily conserved signaling intermediate in Toll pathway (TRAF6-ECSIT) complex, to enhance antibacterial killing effects through mtROS. IFN-γ signaling activates estrogen-related receptor α (ERRα) to enhance antimicrobial clearance through mtROS generation. mtROS are closely related to the pathways of autophagy and inflammasome, both essential in the maintenance of cellular homeostasis and activation of innate host defense. 5′ AMP-activated protein kinase (AMPK) inhibits, but hypoxia-inducible factor (HIF)-1α enhances, the generation of mtROS, thereby influencing innate defense against several pathogenic bacteria. HIF-1α/mTOR signaling drives aerobic glycolysis and reactive oxygen species (ROS) generation; mTOR inhibition leads to the enhancement of mtROS to promote host defense against *Trypanosoma cruzi* infection. AMPK activation by metformin is beneficial in the clearance of intracellular bacterial infection through phosphorylation of AMPK and mtROS generation. In addition, AMPK is the upstream kinase for ERRα-mediated antimicrobial responses during *Mycobacterium tuberculosis* (Mtb) infection. During oxidative stress, NRF2 translocates to the nucleus after dissociation from Keap1 to induce the expression of protective antioxidant genes. Mitochondrial antiviral signaling (MAVS) activation results in the expression of COX5B, which inhibit the mtROS to block the MAVS-mediated antiviral signaling. mtROS involvement in antiviral responses is described in the text in details. **(B)** Uncontrolled mtROS production leads to excessive inflammatory responses, tissue damage, and necrosis, thus detrimental to host defense. Dysregulated activation of NLRP3 inflammasome results in tissue damage and pathological inflammation through mitochondrial dysfunction and mtROS generation. Several host factors [sirtuin 3 (SIRT3), miR-23a, and peroxisome proliferator-activated receptor gamma (PPARγ)] are negative regulators for controlling excessive mtROS generation, thereby coordinating antimicrobial host defense. Excessive ROS production blocks the nuclear translocation and activity of NRF2. The accumulation of mtROS, which is also mediated by sustained endoplasmic reticulum (ER) stress, is detrimental to host defense.

Autophagy, an intracellular homeostatic process during stress conditions (14), contributes to regulating the production of mtROS during viral infection. Previous studies showed that mtROS generation, accompanied by dysfunctional mitochondria, was upregulated to promote RLR-mediated mitochondrial antiviral signaling protein (MAVS/IPS-1) signaling and resistance to vesicular stomatitis virus infection in ATG5-deficient primary mouse embryonic fibroblasts (MEF) cells and macrophages (29). During Sendai virus infection, mitochondrial COX5B, the ETS component of the cytochrome *c* oxidase complex subunit, can interact with MAVS and ATG5 and contributes to the balance in MAVS-mediated antiviral signaling (36). Mechanistically, MAVS activation results in the expression of COX5B, which, in turn, downregulates MAVS-mediated antiviral signaling through the inhibition of mtROS. These data suggest a link between the mitochondrial ETS system and MAVS-mediated antiviral signaling through the modulation of ROS (36). A recent study also showed that Parkin, a key player in mitophagy, plays an inhibitory role in antiviral immunity through controlling the mtROS-NLRP3 inflammasome during viral infection. Parkin deficiency amplifies antiviral inflammation by enhancing mtROS to activate the NLRP3 inflammasome, thereby enhancing viral clearance in macrophages and dendritic cells (37). Taken together, these studies highlight the role of mtROS in the balanced regulation of the autophagy–inflammasome axis, which is crucial for the innate host defense. The protective roles of mtROS in antimicrobial innate immune defense are summarized in Figure 1A.

### Pathogens Modulate mtROS to Promote Pathogenesis and Virulence

#### Bacteria, Fungi, and Parasites Regulate Host mtROS for Their Own Benefits

Several studies showed that pathogens and their components are able to evade host immune system or augment pathological inflammation through modulation of mitochondrial oxidative stresses for their own benefits. A recent study showed that the outer membrane protein 34 (Omp34) of *Acinetobacter baumannii*, a Gram-negative opportunistic pathogen, triggers mtROS generation, leading to the hyper-activation of NLRP3 inflammasome and pyroptosis during infection (38). In addition, *Escherichia coli* O157:H7 can induce severe inflammation through damage of mitochondria and mtROS, which triggered NLRP3 inflammasome activation; this was ameliorated by quercetin *via* prevention of ROS and autophagy activation (39). Moreover, *Aspergillus* protease stimulation of lung epithelial cells (A549 cells) upregulated mRNAs of a variety of inflammatory cytokines and intercellular adhesion molecule (ICAM)-1 through mtROS (40), suggesting the role of mtROS as a therapeutic target for fungal inflammation.

In *Pseudomonas aeruginosa* infection, pyocyanin is an important virulence factor through which mtROS mediate cell apoptosis through the induction of mitochondrial acid sphingomyelinase in neutrophils (41). In addition, pyocyanin can trigger natural killer (NK) cell apoptosis through mitochondrial damage and intracellular calcium release (42). Although the exact role for mtROS was not described in this study, intracellular ROS, generated by the NADPH oxidase, was not involved in NK cell death (42). As mitochondrial damage results in the excessive mtROS production in numerous pathological conditions (43), future studies will clarify the role for mtROS in immune cell death during *P. aeruginosa* infection.

Early studies showed that the intracellular parasite *Leishmania donovani* targets and stabilizes host transcriptional factor SREBP2 to regulate macrophage cholesterol and inhibit microbicidal mtROS production, thereby favoring parasite persistence (18). In another study, *L. donovani* infection induced the upregulation of uncoupling protein 2 (UCP2) to inhibit mtROS generation, thereby attenuating host Th1-biased immune response and parasitic clearance (19). These data support the idea that several pathogens may alter the host gene program and/or immune metabolism to up- or downregulate mtROS production, thereby inhibiting host cell death and/or facilitating parasite survival.

Bacterial pathogens can also inhibit potentially harmful oxidative radicals for their own survival. In human gingival epithelial cells, *Porphyromonas gingivalis* can circumvent the hypochlorous acid (HOCl)-mediated antimicrobial clearance system through the activation of host glutathione synthesis pathways (44). *Porphyromonas gingivalis* nucleoside-diphosphate kinase (Ndk) was found to act as an effector, inhibiting antimicrobial signaling activities by extracellular adenosine triphosphate-induced ROS production, thus leading to pathogen persistence in gingival epithelial cells (45). Overall, the data suggest that several microbial pathogens evade or inhibit the host-defensive mtROS pathway and bactericidal free radicals (Table 1). Future experimental data are needed to elucidate the mechanisms by which each pathogen modulates the components/pathways of the host mtROS production system.

**Table 1:** Pathogen-mediated modulation of mtROS.

**Pathogens**	**Study model**	**mtROS**	**Consequences**	**Mechanism of action**	**References**
**Bacteria**					
*A. baumannii*	RAW264.7 cells	↑	Induction of pyroptosis and apoptosis	Activation of NLRP3 inflammasome	(38)
*E. coli O157:H7*	Human colonic epithelial Caco-2 cells	↑	Induction of severe inflammation	Excessive ROS production from damaged mitochondria leading to NLRP3 inflammasome activation, which is inhibited by quercetin	(39)
*P. aeruginosa*	Primary murine neutrophils, HL-60 cells	↑	Neutrophil death	Pyocyanin-induced activation of neutrophil death through mitochondrial acid sphingomyelinase	(41)
*P. gingivalis*	Human primary gingival epithelial cells	↓	Increased bacterial survival and persistence	Inhibition of eATP/NOX2-ROS-antibacterial pathway	(44)
	Human primary gingival epithelial cells	↓	Increased bacterial survival and persistence	Upregulation of the antioxidant glutathione responses to inhibit eATP-induced cytosolic and mtROS	(45)
**Parasite**					
*L. donovani*	Murine peritoneal macrophages, *in vivo*	↓	Facilitation of parasite entry and survival	SREBP2-dependent upregulation of UCP2, a mitochondrial inner membrane protein, suppresses mtROS generation	(18)
	RAW264.7 cells, murine splenic macrophages, *in vivo*	↓	Establishment of infection; anti-inflammatory immune responses	Upregulation of UCP2 suppresses mtROS; Inactivation of MAPK to ameliorate a Th1-biased immune responses	(19)
*T. cruzi*	C2C12 cell line, human cardiac myocytes, HeLa, *in vivo*	↑	Heart failure in chagasic cardiomyopathy (CCM)	Excessive ROS-dependent inhibition in the nuclear translocation and activity of NFE2L2 (Nrf2) and induction of fibrotic gene expression	(46)
**Virus**					
RSV	A549 cells, vero cells, BCi-NS1 cells, pBECs	↑	Facilitation of viral infection	RSV induces mitochondrial redistribution, impairs mitochondrial respiration, loss of mitochondrial membrane potential	(47)
HIV	Human astrocytes	PI, ↓; NPI, ↑	PI, astrocyte survival; NPI, Pyroptosis	PI, increased autophagic flux and activation of mitophagy; NPI, NLRP3-caspase-1-GSDMD pathway activation	(48)
IAV	*In vivo*; murine alveolar macrophages and neutrophils	↑	Exacerbation of viral pathogenesis	The mechanisms of IAV-mediated induction of mitoROS are not described; mitoTEMPO alleviates lung inflammation and attenuates the death of neutrophils and macrophages.	(49)
	NHNE cells	↑	Restriction of IAV replication	Production of IFN-λ *via* mitochondrial and Duox2-grenerated ROS	(30)
	NHNE cells	↑	Inhibition of IAV viral titer	STAT phosphorylation and induction of IFN-stimulated genes	(31)

*eATP, extracellular adenosine triphosphate; P2X_7_, purinergic receptor; NADPH, nicotinamide adenine dinucleotide phosphate; NOX2, NADPH oxidase 2; NLRP3, NLR family Pyrin domain-containing 3; SREBP2, sterol regulatory element binding protein 2; UCP2, uncoupling protein 2; MAPK, mitogen-activated protein kinase; NFE2L2 (Nrf2), nuclear factor erythroid 2-related factor 2; RSV, respiratory syncytial virus; pBEC, primary human bronchial epithelial cells; HIV, human immunodeficiency virus; PI, productively infected; NPI, nonproductively infected; GSDMD, gasdermin D; IAV, influenza A virus; NHNE, normal human nasal epithelial cells; IFN, interferon; Duox2, dual oxidase 2; STAT, signal transducer and activator of transcription; ↑, increase; ↓, decrease*.

#### Viruses Manipulate Host mtROS for Pathogenesis

Numerous viruses co-opt host mitochondrial functions and mtROS to favor their pathogenesis and virulence (Table 1). A recent study showed that respiratory syncytial virus infection resulted in the impairment of mitochondrial respiration, loss of mitochondrial membrane potential, and increased mtROS, which promoted and favored viral replication in the cells (47). In addition, human immunodeficiency virus (HIV) can suppress or enhance mtROS generation in both productive and non-productive stages for its own benefit. Productive HIV infection in human astrocytes led to the attenuation of mtROS production and dissipation of matrix metalloproteinases, thereby making the virus resistant to cell death, whereas non-productive HIV infection induced mitochondrial damage and inflammasome-induced cell death (48). Mechanistically, productive HIV infection increased the mitophagy to resist cell death, whereas non-productive HIV infection enhanced the pyroptosis of astrocytes through NLRP3-mediated gasdermin D pathway activation (48). Thus, HIV co-opts mtROS and mitochondrial damage in both productive and non-productive infection to drive viral pathogenesis in HIV-associated neurodegenerative diseases (48). In addition, IAV can also circumvent antiviral responses and favor viral replication through NADPH oxidase 2-mediated endogenous ROS generation (50–52). Additional studies are warranted to reveal how a variety of viruses manipulate mtROS in relation to pathological inflammation, thereby enhancing their virulence and pathogenesis.

### Detrimental Roles of mtROS During Infection and Sepsis

#### mtROS in the Amplification of Pathological Responses During Infection

Uncontrolled ROS production is thought to lead to necrosis, thereby resulting in hyper-inflammation and tissue damage (53). Tumor necrosis factor (TNF) is an essential cytokine for the maintenance of host defense during human tuberculosis (TB), as patients with rheumatoid arthritis often develop TB reactivation during anti-TNF therapy (54). In the pathogenesis of TB, mtROS production due to hyper-TNF responses plays a detrimental role, inducing host cell-programmed necrosis and the propagation of mycobacteria into the extracellular milieu (54). In a recent study, sirtuin 3 was shown to be critically involved in the host defense against Mtb infection through the control of exaggerated proinflammatory responses and neutrophil infiltration (55). These protective effects were mediated through the sirtuin 3-dependent inhibition of mitochondrial dysfunction and excessive mtROS generation in macrophages during Mtb infection (55). During infection with *E. coli* O157:H7, a pathogen that causes serious gastrointestinal infection, mitochondrial dysfunction and oxidative stress were associated with excessive inflammation and host damage (39). In addition, exaggerated production of mtROS results in fungus-mediated pathological inflammation in airway epithelial cells (40) and *T. cruzi*-mediated cardiomyopathy in Chagas disease (46). Also, mtROS inhibition suppressed respiratory syncytial virus replication and virus-mediated lung inflammation (47). These data suggest the pathogenic role of mtROS during infections of various pathogens.

As mtROS affect the number of phagocytes during infection, blockade of mtROS or superoxide dismutase functions in beneficial roles through the prevention of cell death in several severe infections. Previous studies showed that either Sod2 deficiency or MitoTEMPO, a scavenger of mtROS, treatment increased neutrophil numbers and reduced the bacterial burden during *P. aeruginosa* infection in zebrafish models (56). Moreover, recent studies showed that IAV-mediated exacerbation of viral pathogenesis is ameliorated by scavenging mitoROS (49). During IAV infection, the local delivery of MitoTEMPO significantly inhibited viral titers and mortality in mice infected with IAV (Hkx31, H3N2) and attenuated apoptotic and necrotic neutrophils/macrophages in lung tissues during infection (49). These data suggest the pathological effects of mtROS that promote harmful inflammation and cell death during severe pathogenic infection and support the potential use of mtROS scavengers as host-directed adjuvant therapies.

#### Host Factors and Mechanisms That Ameliorate mtROS Generation During Infection

There are several reports suggesting the mechanisms by which mtROS generation is controlled in the host cells during infection. Previous studies have shown that microRNA-23a is essential for the maintenance of mitochondrial integrity and restriction of mtROS through the targeting of PPIF, the gatekeeper of mitochondria permeability transition pores, thereby blocking excessive necrosis and liver damage in *L. monocytogenes* infection (53). In rotavirus infection, the accumulation of mtROS is mediated by sustained endoplasmic reticulum (ER) stress and the release of calcium from the ER to the mitochondria, which further contributes to viral inflammation (57). Furthermore, the inhibition of mtROS, at least partly through peroxisome proliferator-activated receptor gamma (PPARγ) activation, played a beneficial role in the reduction of rotavirus infection (58). These data encourage us to target mtROS as a therapeutic strategy for viral infections by alleviation of virus-mediated ER stress and inflammatory responses (57, 58).

There is emerging interest in the role of nuclear factor erythroid 2-related factor 2 (NFE2L2)/Nrf2, a master regulator of antioxidant and anti-apoptosis system (59, 60), in the regulation of mitochondrial homeostatic functions in a variety of physiological and pathological conditions including infection (60, 61). During *P. aeruginosa* infection, the non-histone nuclear protein HMGN2 contributes to the cell-autonomous immune defense through the clearance of pyocyanin-mediated intracellular oxidative stress *via* elevation of the Nrf2-mediated antioxidant gene (62). In *T. cruzi* infection, mtROS production was increased *in vivo* and *in vitro*, resulting in the inhibition of nuclear translocation of Nrf2 and antioxidant gene expression (46). Preserving Nrf2 activity was beneficial for maintenance of cardiac function in heart failure of infectious etiologies (46). Together, these findings highlight the mechanisms by which mtROS accumulation and mitochondrial damage are mediated, although a large body of mechanism has not been well-defined, in a variety of infections. The actions of known host factors to regulate mtROS in the context of infection are illustrated in Figure 1.

#### Detrimental Roles of mtROS During Sepsis

Relatively well-characterized functions of mtROS generation have been shown in the human and animal model of sepsis. In patients with sepsis, mitochondrial function and antioxidant defenses appear to have important roles in the protection against multi-organ failure through the control of harmful excessive ROS production (63, 64). In sepsis-induced acute kidney injury, mitochondrial alteration and ROS production contributed to pathology, which was ameliorated by SIRT3, a NAD(+)-dependent deacetylase (65). In a study, mesenchymal stromal cells showed beneficial effects on experimental sepsis through the inhibition of macrophage NLRP3 inflammasome activation *via* suppression of mtROS (66). A recent genetic study showed that a specific mtDNA mutation (T6459C) might be associated with genetic susceptibility to sepsis, as the mutation group showed increased ROS levels and apoptosis (67). GABA type A receptor-associated protein (GABARAP) deficiency led to increased susceptibility to sepsis through the enhancement of mtROS and proinflammatory cytokine expression with NLRP3 inflammasome activation (68). Furthermore, blockade of the NOTCH pathway inhibited mtROS generation through the suppression of glucose oxidation, thereby attenuating hepatic macrophage M1 shifting, which reduced the lethality of endotoxin-mediated fulminant hepatitis (69). These data suggest that understanding the molecular mechanisms by which host factors target mtROS generation can contribute to the development of potential therapeutic strategies against sepsis.

Given the role of mtROS in the pathogenesis of sepsis, targeted delivery of antioxidants to mitochondria has been suggested as a potential therapeutic strategy against sepsis (63). Glucocorticoids, which are widely used anti-inflammatory drugs, control endotoxin-mediated inflammation and sepsis by inhibiting mitochondrial calcium homeostasis and the production of mtROS (70). Stefin B, an endogenous cysteine cathepsin inhibitor, was found to be essential for the control of LPS-induced sepsis through the stabilization of mitochondrial membrane potential and amelioration of mtROS generation (71). In a cecal ligation and puncture mouse model of sepsis, synthetic antioxidant lignan secoisolariciresinol diglucoside (SDG; LGM2605) alleviated septic cardiac dysfunction. Mechanistically, LGM2605 improved cardiac function by protecting cardiac mitochondrial function and inhibiting ROS accumulation (72). Furthermore, maresin 1, a metabolite of the omega-3 fatty acid, had significant inhibitory effects on mtROS generation but increased ATP content and the mtDNA copy number, thereby showing a protective role against sepsis (73). Taken together, these data suggest that the agents and small molecules involved in the control of mitochondrial dysfunction could attenuate pathological responses during sepsis through the amelioration of mtROS. Future studies for the pharmacological approaches targeting mtROS may develop potentially promising strategies to increase survival and treatment efficacy of sepsis.

## Conclusion

The past several years have provided us with increasing evidence of the means by which the mtROS signaling pathway affects innate immunity during infection. mtROS generation *via* pathogen infection may bring together the intracellular autophagy, immune, and metabolic pathways to guide and shape the effector response. mtROS-mediated antiviral signaling is linked partially to MAVS and STAT signaling, leading to the antiviral host defense. In addition, mtROS is a crucial signal for bactericidal responses and NLRP3 inflammasome activation to enhance antibacterial responses. Nevertheless, our current knowledge is limited on how mtROS activates antibacterial or antiviral responses, and how mtROS are implicated in the inflammatory signaling pathways under different pathogenic stimuli. Further studies to understand molecular mechanisms will shed new light on the impacts of the mtROS signaling network on distinct innate effector functions and/or for driving pathological inflammatory responses during infection.

Moreover, a variety of pathogens modulate, escape, or enhance mtROS production for their own benefit, although our understanding of the underlying mechanisms remains limited. In the future, elucidating how pathogens interact with host-defensive machineries of mtROS system should help to assess the pathogenesis and/or defensive pathways in the context of infection. As mtROS generation is interconnected with the immunometabolic status in macrophages, it would be extremely interesting to investigate how mtROS crosstalk with the AMPK-mTOR pathway during infection. Further exploiting the host-defensive mechanisms for repressing pathological mtROS production and function is a crucial next step in developing therapeutic application. Although accumulating data have provided extensive evidence that mtROS contribute to inflammation-induced pathology during sepsis, therapeutic outcomes have been limited in the use of mtROS modulators in clinical settings.

Many of the yin-and-yang aspects of mtROS signaling remain unknown in terms of infection. Answers to the above questions will provide exciting new opportunities for therapeutic approaches to many diverse infections.

## Author Contributions

E-KJ: designed. E-KJ, PS, JK, and YK wrote and reviewed the manuscript. PS: summarized the table. JK and YK prepared the figure. All authors read and approved the final version of the review.

## Conflict of Interest

The authors declare that the research was conducted in the absence of any commercial or financial relationships that could be construed as a potential conflict of interest.
